# Actions of Hydrogen Sulfide on Sodium Transport Processes across Native Distal Lung Epithelia (*Xenopus laevis*)

**DOI:** 10.1371/journal.pone.0100971

**Published:** 2014-06-24

**Authors:** Alexandra Erb, Mike Althaus

**Affiliations:** Institute of Animal Physiology, Justus-Liebig University of Giessen, Giessen, Germany; University of Giessen Lung Center, Germany

## Abstract

Hydrogen sulfide (H_2_S) is well known as a highly toxic environmental chemical threat. Prolonged exposure to H_2_S can lead to the formation of pulmonary edema. However, the mechanisms of how H_2_S facilitates edema formation are poorly understood. Since edema formation can be enhanced by an impaired clearance of electrolytes and, consequently, fluid across the alveolar epithelium, it was questioned whether H_2_S may interfere with transepithelial electrolyte absorption. Electrolyte absorption was electrophysiologically measured across native distal lung preparations (*Xenopus laevis*) in Ussing chambers. The exposure of lung epithelia to H_2_S decreased net transepithelial electrolyte absorption. This was due to an impairment of amiloride-sensitive sodium transport. H_2_S inhibited the activity of the Na^+^/K^+^-ATPase as well as lidocaine-sensitive potassium channels located in the basolateral membrane of the epithelium. Inhibition of these transport molecules diminishes the electrochemical gradient which is necessary for transepithelial sodium absorption. Since sodium absorption osmotically facilitates alveolar fluid clearance, interference of H_2_S with the epithelial transport machinery provides a mechanism which enhances edema formation in H_2_S-exposed lungs.

## Introduction

Hydrogen sulfide (H_2_S), a gas with the typical smell of rotten eggs, is well known as a highly toxic environmental chemical threat. The high toxicity of H_2_S is, amongst other things, related to the fact that it is inhaled and can easily cross the alveolo-capillary barrier and is rapidly distributed within the human body [1]. The pulmonary epithelium is the first cell layer which is exposed to H_2_S. Although epithelial cells have been recently shown to have a high capacity to metabolize H_2_S [2], exposure to high concentrations of H_2_S (>500 ppm) can lead to severe lung irritation [3]. Furthermore, prolonged exposure to H_2_S (250–500 ppm) can impair the barrier function of the alveolar epithelium and lead to the formation of pulmonary edema in humans [3]. Similarly, pulmonary edema occurs in mice after exposure to H_2_S concentration >50 ppm [4].

However, the cellular and molecular mechanisms of how prolonged H_2_S exposure promotes edema formation are not completely understood.

The formation of pulmonary edema occurs due to infiltration of fluid into alveoli [5]. Furthermore, edema is enhanced due to an impaired resolution of fluid across the alveolar epithelium (alveolar fluid clearance) [5].

Alveolar fluid clearance is driven by an osmotic gradient across the alveolar epithelium. This osmotic gradient is created by active absorption of ions – particularly Na^+^ ions – across the epithelial cells [6]. The molecular nature of Na^+^ transport has been discovered in pioneer studies by Ussing and colleagues using frog skin preparations [7]: Na^+^ ions enter epithelial cells at the apical membrane via Na^+^-selective ion channels, such as the epithelial Na^+^ channel (ENaC). These Na^+^ ions are actively pumped out of the cells, in exchange for K^+^, due to the activity of the Na^+^/K^+^-ATPase at the basolateral membrane of the epithelium. This vectorial net movement of Na^+^ ions osmotically drives alveolar fluid clearance and thus prevents pulmonary edema.

On the other hand, impaired transepithelial Na^+^ transport is associated with the formation of pulmonary edema [5], [8], [9]. Transgenic mice which have a constitutively decreased pulmonary Na^+^ transport (due to reduced ENaC expression) are predisposed to edema formation [10]. Furthermore, the development of pulmonary edema in patients with *acute lung injury* (ALI) or its more severe form *acute respiratory distress syndrome* (ARDS) is tightly connected to impaired transepithelial Na^+^ absorption and patients with a functional Na^+^ transport have a better clinical outcome [11].

Given the association between edema formation and impaired absorption of Na^+^ ions across the alveolar epithelium, we hypothesized that H_2_S may affect the Na^+^ transporting machinery in pulmonary epithelial cells. We have recently shown that exogenously applied H_2_S decreases Na^+^ transport across a monolayer of a pulmonary epithelial cell line (H441) [12]. However, these data need to be confirmed in more physiologically relevant models. Unfortunately, the complex anatomy of a mammalian lung does not allow the preparation of native distal (i.e. alveolar) lung epithelia for transepithelial transport studies in Ussing chambers. The use of primary isolated alveolar epithelial cells and cell lines can also be problematic since the expression of ion channels which are required for vectorial Na^+^ transport is highly dependent on cell culture conditions [13], [14]. For those reasons we performed transepithelial transport studies on freshly isolated amphibian lungs from *Xenopus laevis*. These lungs have a simple sac-like anatomy and can easily be dissected into flat sheets which are suitable for Ussing chamber recordings. *Xenopus* lungs have only one epithelial cell type (pneumocyte) which is homologous to mammalian alveolar epithelial cells and has features of both alveolar type I and type II cells [15]. These pneumocytes express functional ENaCs as well as the Na^+^/K^+^-ATPase and have an ion channel and transporter repertoire which is similar to those of mammals [16], [17], [18], [19], [20].

Using this model, we demonstrate that exposure of these native lung epithelia to H_2_S decreases the rate of Na^+^ absorption due to an impairment of the Na^+^/K^+^-ATPase as well as K^+^ channels in the basolateral membrane of pulmonary epithelial cells.

## Materials and Methods

### Administration of H_2_S

**Figure 1:**
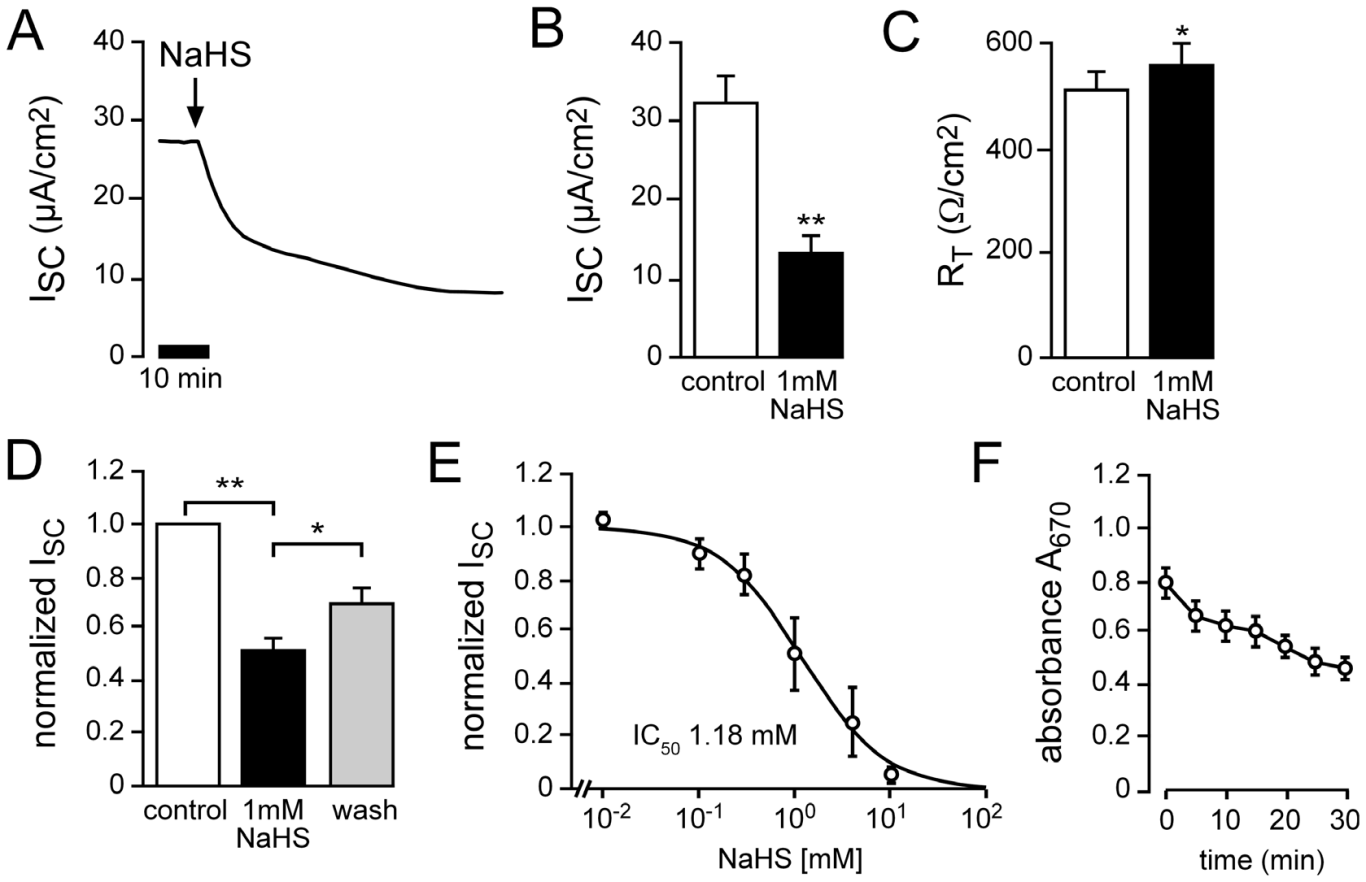
Exogenous H_2_S inhibits net ion transport of *Xenopus* lung epithelia. **A**) Representative current trace of an Ussing chamber recording. The application of NaHS (1 mM) to the apical compartment of the chamber led to a strong decrease in transepithelial ion current (I_SC_). **B**) Statistical analysis of experiments as shown in panel A. NaHS significantly reduced I_SC_ by approx. 60% (n = 19, N = 13, p≤0.01). **C**) Following application of NaHS, transepithelial resistance (R_T_) increased significantly (n = 19, N = 13, p≤0.05). **D**) The effect of NaHS was partially reversible. Depicted are values of I_SC_ which were normalized to baseline values before application of NaHS. After wash-out of NaHS, there was a significant increase in current (n = 8, N = 5, p≤0.05). **E**) NaHS dose-dependently decreased I_SC_ (n = 2–3, N = 3). Data were obtained from Ussing chamber recordings in which cumulative doses of NaHS were applied. Total values of I_SC_ were fitted according to the Hill equation. **F**) Evaporative loss of H_2_S during experiments. NaHS (1 mM) was applied to NRS and aliquots were taken every 5 min. H_2_S was indirectly measured by the formation of methylene blue and its absorption at 670 nm (n = 3).

H_2_S was administrated by the donor molecule NaHS (Sigma, Taufkirchen, Germany). NaHS dissociates in aqueous solution into Na^+^, H_2_S/HS^−^ and OH^−^. To avoid unspecific side effects due to alterations in pH, buffer solutions were adjusted to keep the pH values stable over the time course of experiments (data not shown). Initially, a concentration-response experiment was performed (Figure 1 E) and the IC_50_ value for transport inhibition by NaHS was determined as 1.18 mM. This corresponds to ∼50 ppm and is in a concentration range which is associated with lung irritation and pulmonary edema [4], [21]. Therefore, 1 mM was employed as the standard concentration for NaHS.

### Determination of H_2_S in buffer solutions

In order to monitor the evaporative loss of H_2_S from the employed buffer solutions (see below), H_2_S concentrations where measured indirectly by the formation of methylene blue as previously described [22]. Aliquots (300 µl) were taken every 5 min after preparation of a solution containing 1 mM NaHS over a time period of 30 min. Aliquots were immediately mixed with ice-cold 4% zinc acetate and incubated for 30 min on ice. Afterwards, 200 µl of 0.1% dimethylphenylendiamine sulfate (DMPPDA; in 5 M HCl) and 100 µl of 50 mM FeCl_3_ (in 1.2 M HCl) were added to the samples. Samples were centrifuged at 5000×g at room temperature for 10 min and incubated for another 5 min at room temperature. The amount of formed methylene blue corresponds to the concentration of H_2_S in the buffers. Samples were eventually diluted 1∶10 and methylene blue was measured as absorbance at 670 nm with a spectrophotometer (Krüss Optronic, Germany).

### Animal treatment and tissue preparation

Due to the complex anatomy of the mammalian lung it is not possible to perform electrophysiological measurements of ion transport across a native distal (alveolar) lung epithelium in rats or mice. Therefore, South-African clawed frogs (*Xenopus laevis*) were used as a well-established vertebrate model for pulmonary ion transport physiology [16], [17], [18], [19], [20]. Adult clawed frogs were purchased from Xenopus Express France (Vernassal, France) and kept in tanks with continuous freshwater supply. For experiments, frogs were tranquilized in ice water and subsequently killed by decapitation and sounding of the spinal cord. Lungs were dissected and longitudinally opened to flat sheets by incision from the bronchi along the pulmonary artery. Lung sheets were subsequently mounted between Lucite rings and transferred to perfusion Ussing chambers. The treatment of animals conformed to the German law of animal care and was authorized by the regional board of Giessen (“400_M Xenopus laevis”).

### Ussing chamber studies

The excised lung sheets which were fixed between Lucite rings were mounted into custom-made perfusion Ussing chambers. The sheets separated the chamber halves into an apical and a basolateral compartment, which thereby allowed the specific application of drugs to either side of the epithelium. The chamber halves were perfused separately by a gravity-driven perfusion system. The chambers were perfused with neutral Ringer's solution (NRS), which contained (in mM): 100 NaCl, 3 KCl, 1 CaCl_2_, 1 MgCl_2_, 10 4-(2-hydroxyethyl)-1-piperazineethanesulfonic acid (HEPES) and 10 glucose (pH 7.4, Tris-base).

The chambers were connected to a voltage-clamp amplifier by Ag/AgCl electrodes which were mounted into the chambers via 200 µl pipette tips which were filled with 3% 1 M KCl-agar (Sigma, Taufkirchen, Germany). Only those electrodes were employed, which had a spontaneous electrical potential of less than 1 mV in perfusion solution. After complete mounting of the tissue and electrodes, the perfusion was started and the transepithelial potential (V_T_) was monitored. After equilibration of V_T_, the tissues were clamped to 0 mV and short-circuit currents (I_SC_) were recorded on a PC via an analog/digital interface and the software LabScribe (National Instruments, Munich, Germany). I_SC_ values were additionally recorded on a strip-chart recorder (Kipp&Zonen, Delft, The Netherlands). In order to determine the transepithelial resistance (R_T_), 5 mV voltage pulses were applied to the epithelia and the resulting current deflections were recorded. R_T_ was then calculated following Ohm's law. All experiments were performed at room temperature.

### Measurement of basolateral membrane conductance

In order to measure the activity of the Na^+^/K^+^-ATPase, apical membranes were permeabilized with nystatin (100 µM, Sigma). The use of nystatin for the specific permeabilisation of apical or basolateral membranes is a well-established method in epithelial physiology [23]. Nystatin generates pores which are selective for Na^+^, K^+^ and Cl^−^ [24]. It does not cross the cell and permeabilise the opposite (basolateral) membrane [23]. Amiloride, an inhibitor of ENaC, was also present in the apical solution (10 µM). Under these conditions, I_SC_ values reflect currents across the basolateral membrane, which are mainly due to Na^+^/K^+^-ATPase activity.

In order to measure basolaterally located K^+^ channels, the apical side of the epithelium was perfused with a high K^+^ NRS which contained (in mM): 3 NaCl, 100 KCl, 1 CaCl_2_, 1 MgCl_2_, 10 HEPES and 10 glucose (pH 7.4, Tris-base). This resulted in an apical to basolateral K^+^ gradient (100∶3). After equilibration of the I_SC_, ouabain (1 mM) was applied to the basolateral compartment in order to block the Na^+^/K^+^-ATPase and the apical membrane was subsequently permeabilized with nystatin (100 µM). The resulting I_SC_ represented current fluxes via basolateral K^+^ channels.

### Chemicals

Amiloride (Sigma) was used as an inhibitor for ENaC and ouabain (Sigma) in order to block the Na^+^/K^+^-ATPase. Lidocaine (Sigma) was used as an unselective inhibitor of K^+^ channels. Where necessary, dimethyl sulfoxide (DMSO) was used as a solvent and was present in according control experiments.

### Data analysis and statistics

Data are presented as means ± standard error of the mean (SEM). The number of experiments is indicated with “n”, the number of donor frogs is indicated with “N”. For statistical analysis of dependent experiments (e.g. current values before and after drug application), paired Student's t-Test was employed. For independent experiments (e.g. comparing different lung preparations) unpaired Student's t-test was used. In general, a value of p≤0.05 was regarded to be statistically significant and marked with asterisks (* p≤0.05; ** p≤0.01).

## Results

### H_2_S inhibits net ion transport across *Xenopus* lung epithelia

The administration of the H_2_S donating molecule NaHS (1 mM, apical side) decreased transepithelial ion current (I_SC_) across *Xenopus* lung epithelia by approx. 60% (Figure 1 A, B). There was no decrease in transepithelial resistance (R_T_; Figure 1 C) which indicates that the NaHS-mediated decrease in I_SC_ was not the result of damage to the epithelium. Furthermore, the effect of NaHS was partially reversible, since normalized values of I_SC_ increased after wash-out of NaHS (Figure 1 D). The inhibition of ion transport by NaHS was also dose-dependent, with an IC_50_ of 1.18 mM (Figure 1 E). Importantly, in the employed perfusion Ussing chamber system there will be diffusion of H_2_S into the air. The amount of H_2_S was measured over time in the employed Ussing chamber solution by a colorimetric assay and the formation of methylene blue (Figure 1 F). There was a slow but constant decline of buffer H_2_S concentrations within the time frame of experiments which indicates that the effective H_2_S concentration is likely lower than reflected in the determined IC_50_ value for NaHS.

Taken together, these data indicate that exogenously applied H_2_S decreases net ion transport across *Xenopus* lung epithelia in toxicologically relevant concentrations (1 mM NaHS corresponds to ∼50 ppm).

### H_2_S inhibits amiloride-sensitive Na^+^ absorption

**Figure 2:**
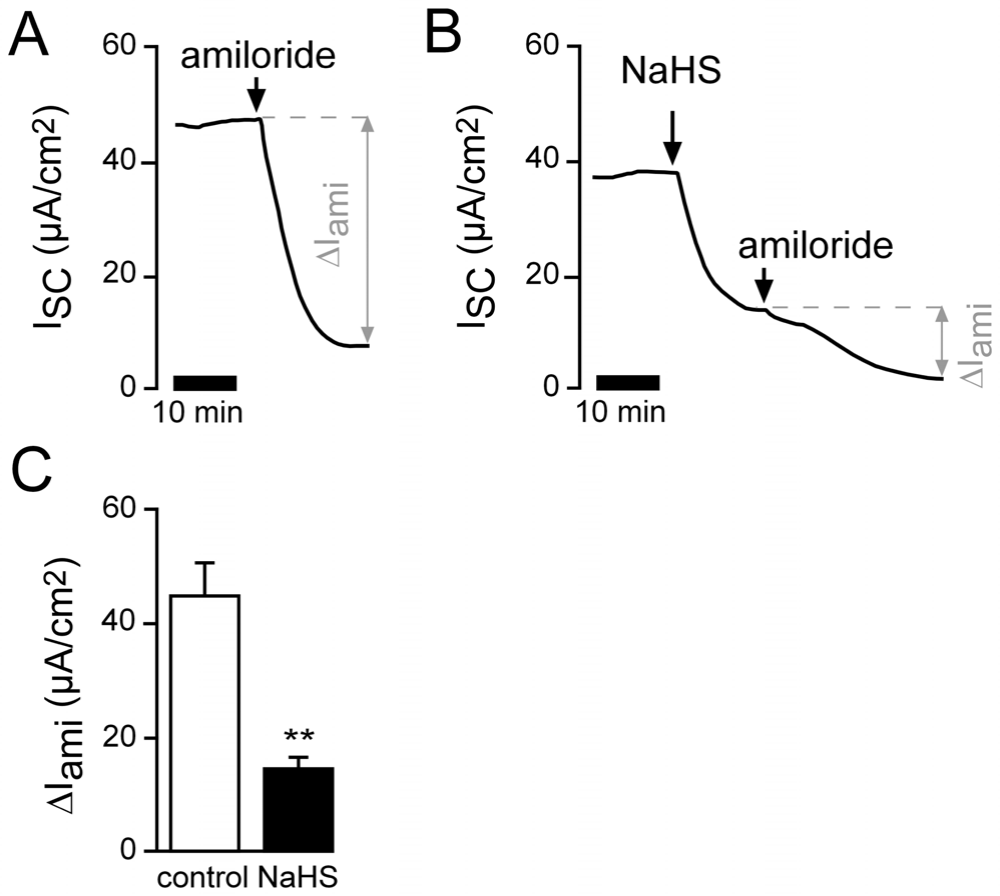
H_2_S inhibits amiloride-sensitive Na^+^ absorption. **A**) Representative current trace of a control experiment. In order to estimate the amount of Na^+^ absorption, the ENaC inhibitor amiloride (10 µM) was applied apically. **B**) Similar experiment showing the effects of amiloride after apical pre-treatment of the lung epithelium with apical NaHS (1 mM). **C**) Statistical evaluation of experiments as shown in panels A and B. Depicted are amiloride-sensitive (ΔI_ami_) current fractions. NaHS significantly reduced ΔI_ami_ from 45.04±5.55 µA/cm^2^ to 14.84±1.77 µA/cm^2^ (n = 5; N = 3; p≤0.001).

Under basal conditions, the main ion transport process in *Xenopus* lung epithelia is the absorption of Na^+^ ions from the apical to the basolateral side via the epithelial Na^+^ channel (ENaC) and the Na^+^/K^+^-ATPase [17], [18], [19], [20]. Therefore it was investigated if Na^+^ absorption was affected by NaHS. In order to address this question, amiloride was employed as an inhibitor of ENaC (Figure 2). Pre-treatment of the lungs with 1 mM NaHS significantly decreased amiloride-sensitive Na^+^ absorption (Figure 2 B, C) compared to control experiments without NaHS (Figure 2 A).

**Figure 3:**
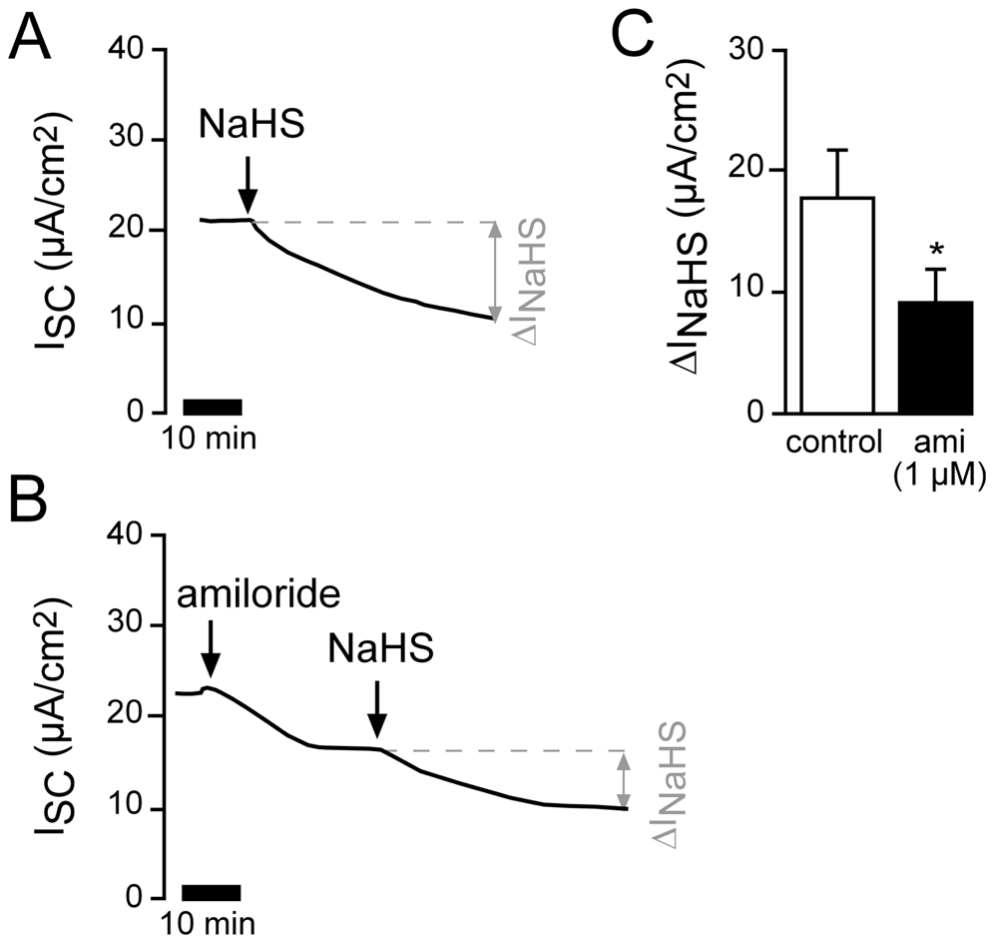
Amiloride attenuates the H_2_S induced current decrease. **A**) Representative current trace of a control experiment showing the effect of apical treatment with NaHS (1 mM). **B**) Lungs were treated with 1 µM amiloride apically and NaHS (1 mM) was subsequently applied for the same duration as the parallel conducted control experiment as depicted in panel A. **C**) Statistical evaluation. Amiloride significantly reduced the NaHS-mediated current decrease (ΔI_NaHS_; n = 6, N = 6, p≤0.05).

In order to confirm this finding, experiments were performed with even more specific concentrations of amiloride (1 µM in the reverse order of drug application (Figure 3)). Following application of 1 µM amiloride, the NaHS-mediated current decrease (ΔI_NaHS_) was significantly smaller than under control conditions. These data indicate that exogenously applied H_2_S impairs amiloride-sensitive, ENaC-mediated Na^+^ transport in *Xenopus* lung epithelial cells.

### Effects of exogenous H_2_S on Na^+^/K^+^-ATPase and basolateral K^+^ channels

A significant contribution to total amiloride-sensitive Na^+^ transport comes from transporting molecules located in the basolateral membrane of epithelial cells. The Na^+^/K^+^-ATPase actively transports Na^+^ out of the cells and thus establishes the chemical gradient for Na^+^ influx via ion channels at the apical membrane. In addition, basolateral K^+^ channels largely determine electrochemical gradients since they contribute to the membrane potential (also of the apical membrane) as well as recycle K^+^ ions which are taken up by the Na^+^/K^+^-ATPase. An inhibition of both, the Na^+^/K^+^-ATPase as well as basolateral K^+^ channels, will eventually decrease total amiloride-sensitive I_SC_ indirectly.

**Figure 4:**
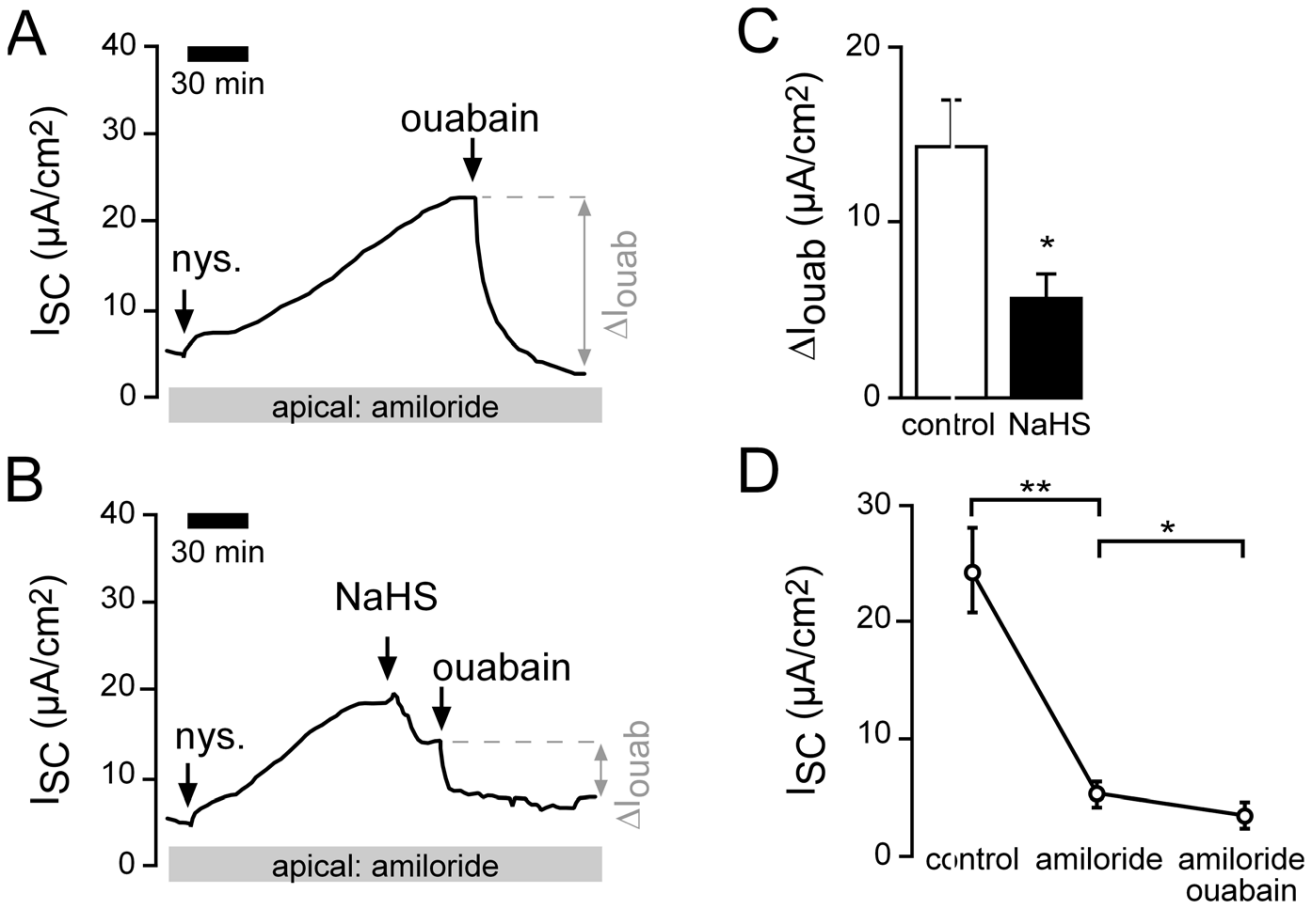
H_2_S decreases Na^+^/K^+^-ATPase currents of *Xenopus* lung epithelia. **A**) Representative current trace of a control recording. The apical membrane of the lung epithelium was permeabilized with nystatin (100 µM, apical) in the presence of amiloride (10 µM). This resulted in a current increase. When the current was stable, the Na^+^/K^+^-ATPase inhibitor ouabain (1 mM) was applied to the basolateral side. The ouabain-sensitive current fraction (ΔI_ouab_) represents the activity of the Na^+^/K^+^-ATPase. **B**) After permeabilisation with nystatin, NaHS (1 mM) was applied to the apical bath. I_SC_ decreased significantly from 20.83±3.99 µA/cm^2^ to 16.17±5.23 µA/cm^2^ (n = 6, N = 6, p≤0.05). Subsequently, ouabain-sensitive current fractions were determined. **C**) Statistical evaluation of experiments as shown in panels A and B. NaHS significantly inhibited Na^+^/K^+^-ATPase activity (ΔI_ouab_; n = 6, N = 6, p≤0.05). **D**) Without permeabilisation with nystatin, ouabain had only a minor effect on transepithelial ion current in the presence of amiloride. Depicted are mean values of non-permeabilized lung epithelia which have been treated with amiloride (10 µM, apical) followed by ouabain (1 mM, basolateral).

In order to measure currents across the basolateral membrane, amiloride was added to the apical side of lung epithelia and the apical membrane was subsequently permeabilized with nystatin (100 µM). This resulted in an increase of the I_SC_ (Figure 4). This current is mainly mediated by the activity of the Na^+^/K^+^-ATPase, since the perfusion solutions do not generate ion gradients across the permabilized epithelium, which would allow ion fluxes through basolateral ion channels. Furthermore, the nystatin-induced current was fully sensitive to the Na^+^/K^+^-ATPase inhibitor ouabain (Figure 4 A), whereas without nystatin permeabilisation and in the presence of amiloride, ouabain had only a minor effect on I_SC_ (Figure 4 D). When NaHS was applied to the apical bath after permeabilisation with nystatin, I_SC_ decreased significantly (Figure 4B). The ouabain-sensitive fractions of the current (ΔI_ouab_) also decreased significantly (Figure 4C), which indicates that the Na^+^/K^+^-ATPase is inhibited by NaHS.

**Figure 5:**
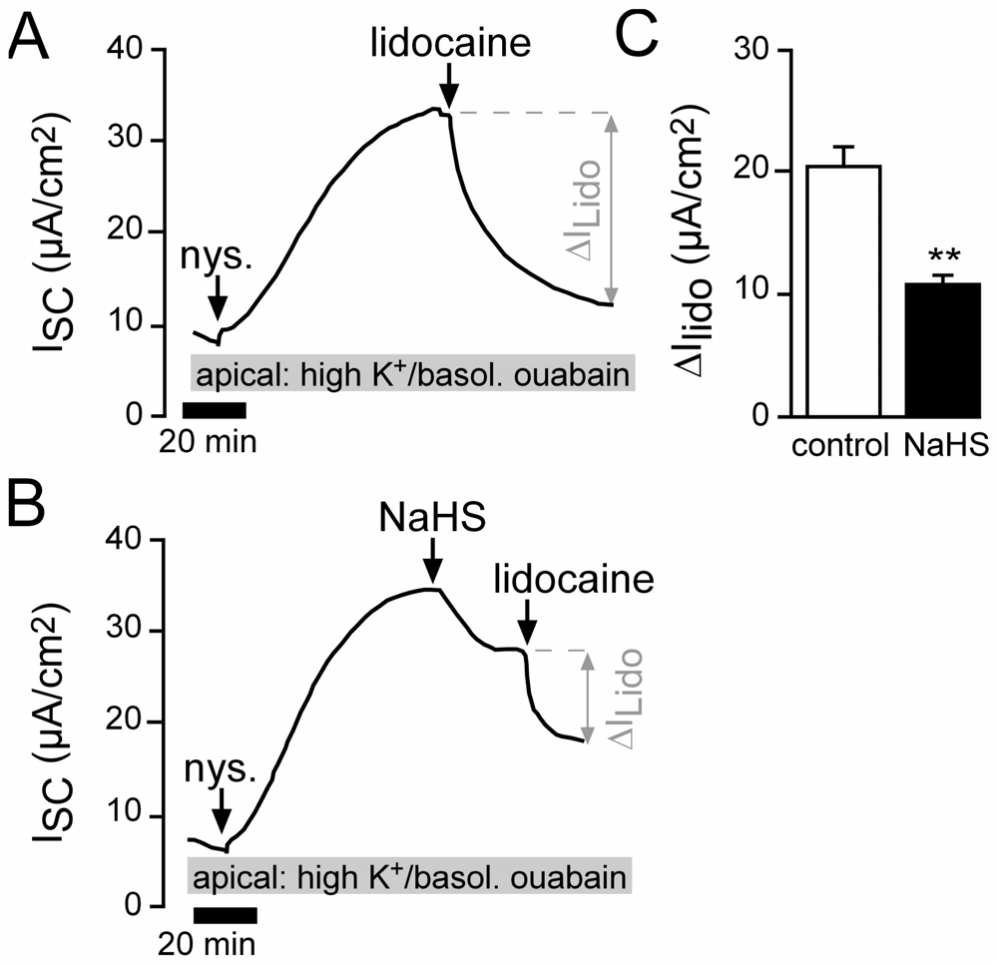
H_2_S inhibits basolateral K^+^ channels. **A**) Representative current trace of a control recording. In order to measure basolateral K^+^ channels, lungs were apically perfused with a high K^+^ solution. Ouabain (1 mM) was present in the basolateral perfusate in order to exclude a contribution of the Na^+^/K^+^-ATPase. Under these conditions, the apical membrane was permeabilized with nystatin (100 µM). This resulted in a current increase which was sensitive to the nonselective K^+^ channel inhibitor lidocaine (1 mM). **B**) The application of NaHS (1 mM) after nystatin permeabilisation resulted in a current decrease (from 47.2±5.12 µA/cm^2^ to 36.4±2.93 µA/cm^2^; n = 5, N = 5, p≤0.05). Subsequently applied lidocaine had a smaller effect compared to control recordings as shown in panel A. **C**) Statistical evaluation of experiments as shown in panels A and B. NaHS significantly reduced lidocaine-sensitive currents (ΔI_Lido_) of the basolateral membrane (n = 5, N = 5, p≤0.01).

In order to measure a putative impact of NaHS on basolateral K^+^ channels, apical membranes of lung epithelia were permeabilized with nystatin (100 µM) in the presence of ouabain (1 mM, basolateral) and an apical to basolateral K^+^ gradient (Figure 5). Under these conditions, the nystatin-induced current was sensitive to the unselective K^+^ channel inhibitor lidocaine (1 mM; Figure 5 A). When NaHS was applied upon nystatin-permeabilisation, I_SC_ decreased significantly (Figure 5 B). Furthermore, NaHS reduced the effect of lidocaine: Lidocaine-sensitive currents (ΔI_Lido_) were significantly smaller compared to those under control conditions (Figure 5 C). These data indicate that NaHS inhibits lidocaine-sensitive K^+^ channels at the basolateral membrane of *Xenopus* lung epithelia.

### Interplay of basolateral K^+^ channels and the Na^+^/K^+^-ATPase

**Figure 6:**
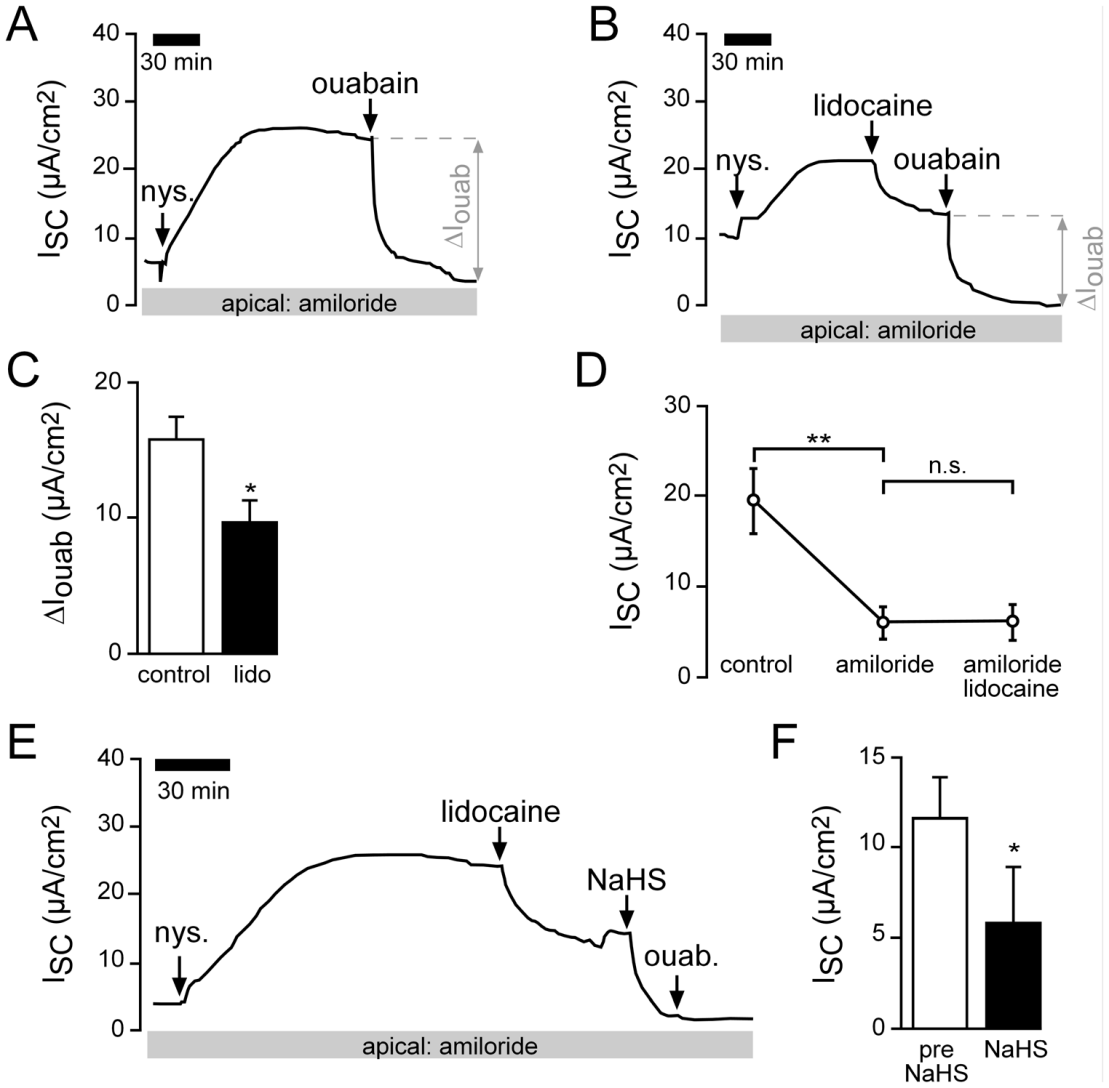
Interplay of basolateral K^+^ channels and the Na^+^/K^+^-ATPase. **A**) Representative current trace of a control recording. The apical membrane of the lung epithelium was permeabilized with nystatin (100 µM, apical) in the presence of amiloride (10 µM). This resulted in a current increase. When the current was stable, the Na^+^/K^+^-ATPase inhibitor ouabain (1 mM) was applied to the basolateral side. The ouabain-sensitive current fraction (ΔI_ouab_) represents the activity of the Na^+^/K^+^-ATPase. **B**) After permeabilisation with nystatin, lidocaine (1 mM) was applied to the basolateral bath. This resulted in a decrease of the I_SC_ from 17.20±2.58 µA/cm^2^ to 12.00±1.67 µA/cm^2^ (n = 5, N = 5, p≤0.05). Subsequently, ouabain-sensitive current fractions were determined. **C**) Statistical evaluation of experiments as shown in panels A and B. Lidocaine (lido.) significantly inhibited Na^+^/K^+^-ATPase activity (ΔI_ouab_; n = 5, N = 5, p≤0.05). **D**) Without permeabilisation with nystatin, lidocaine had only a minor effect on transepithelial ion current. Depicted are mean values of non-permeabilized lung epithelia which have been treated with amiloride (10 µM, apical) followed by lidocaine (1 mM, basolateral). **E**) The application of NaHS (1 mM) to nystatin-permeabilized lungs which have been pre-treated with lidocaine (1 mM) additionally decreased ion current. Note that the subsequent application of ouabain was without any further effect. **F**) NaHS significantly decreased I_SC_ under conditions as shown in panel E (n = 6, N = 6, p≤0.05).

Since NaHS inhibited basolateral K^+^ channels, it is important to investigate if this effect might also be apparent when determining Na^+^/K^+^-ATPase activity on apically permeabilized epithelia. When lidocaine (1 mM, basolateral) was applied to apically permeabilized lung epithelia (Figure 6), a decrease of I_SC_ (Figure 6 B) was observed. Furthermore, ΔI_ouab_ decreased significantly (Figure 6). Without permeabilisation, basolateral lidocaine had no significant effect on I_SC_ (Figure 6 D), which demonstrates that the observed current inhibition is not related to K^+^ channel activity of incompletely permeabilized cells. Together, those data demonstrate that under apically permeabilized conditions, there is still a direct or indirect contribution of a K^+^ conductance to ouabain-sensitive Na^+^/K^+^-ATPase currents. However, when this conductance was blocked with lidocaine (1 mM, basolateral), NaHS still decreased the remaining I_SC_ (ΔI_ouab_) (Figure 6 E, F), which represents an inhibition of Na^+^/K^+^-ATPase currents.

In sum, these data demonstrate that exogenously applied H_2_S inhibits Na^+^ transport across *Xenopus* lung epithelia by inhibiting both the activity of the Na^+^/K^+^-ATPase as well as basolateral, lidocaine-sensitive K^+^ channels.

## Discussion

The formation of pulmonary edema is a hallmark of patients who were exposed to H_2_S – particularly after prolonged exposure [3]. However, the molecular mechanisms leading to edema formation are poorly understood. In the present study we hypothesized that H_2_S may interfere with the clearance of Na^+^ and, consequently, liquid across the pulmonary epithelium. In native *Xenopus* lung preparations, the apical administration of the H_2_S liberating molecule NaHS led to a partially reversible and dose-dependent decrease in net ion transport (Figure 1). The employed concentrations of NaHS as well as the determined IC_50_ value for transport inhibition are within a concentration range (1 mM NaHS corresponds to ∼50 ppm) in which irritation of the respiratory tract [21]as well as pulmonary edema [4] occur. However, it has to be mentioned that due to the employed perfusion Ussing chambers and diffusion of H_2_S into the air, there is a decrease in the absolute H_2_S concentrations over time (Figure 1 F). This indicates that the effective H_2_S concentrations are probably even lower than reflected in the determined IC_50_ value for NaHS.

The observed net decrease in ion transport was due to an impaired amiloride-sensitive Na^+^ transport (Figures 2 and 3). Experiments on functionally isolated basolateral membranes of *Xenopus* lung epithelia demonstrate that the Na^+^/K^+^-ATPase as well as basolateral K^+^ channels are general targets for exogenous H_2_S (Figures 4–6). These findings confirm previous observations using a human airway epithelial cell line (H441; [12]). Both targets are crucial for the maintenance of epithelial Na^+^ transport: the Na^+^/K^+^-ATPase creates the chemical gradients for Na^+^ influx into the epithelial cells via Na^+^ channels located at the apical membrane, whereas basolateral K^+^ channels determine the epithelial membrane potential as well as recycle the K^+^ ions which are taken up by the Na^+^/K^+^-ATPase in exchange for Na^+^ [7].

Impairment of both components, as observed due to H_2_S administration, will eventually decrease the net absorption of Na^+^ ions. Nevertheless, H_2_S might also interact with Na^+^ channels located in the apical membrane. Due to the complex anatomy of the basolateral compartment of the *Xenopus* lung we were never able to get a sufficient permeabilisation of the basolateral epithelial membrane, which would allow the measurement of apically located Na^+^ channels. However, H_2_S has no effect on Na^+^ channels in H441 cells and on heterologously expressed human ENaCs [12]. Nevertheless, a possible effect of H_2_S on Na^+^ channels in the *Xenopus* lung cannot be completely excluded. Irrespective of whether or not Na^+^ channels are affected, the Na^+^/K^+^-ATPase is the major regulator of Na^+^ gradients and its impairment by H_2_S will eventually decrease overall Na^+^ absorption [9]. Consequently, the molecular driving force for liquid clearance is impaired and H_2_S-exposed lungs are predisposed to the formation of pulmonary edema.

An interesting question arises related to the chemical basis for the observed effects of H_2_S on K^+^ channels as well as the Na^+^/K^+^-ATPase. Given a pH value of the employed buffer solution of 7.4, a pK value for H_2_S of ∼7.0 and the assumption that all of the 1 mM NaHS stays in solution (which due to evaporation is not the case; Figure 1 F), the solution would contain ∼286 µM of H_2_S and ∼714 µM HS^−^. This would indicate that HS^−^ represents the main species. In fact, it has recently been demonstrated that the chemical basis for H_2_S-mediated effects on proteins may include the generation of persulfides due to addition of HS- to thiolate or a sulfenic-group; or the interaction of HS^−^ with reactive nitrogen species [25]. Interestingly, in the *Xenopus* lung preparation, NaHS had effects at the basolateral membrane although it was solely applied to the apical compartment. If HS^−^ was not transported into the pulmonary epithelial cells by yet unidentified mechanisms, this would suggest a membrane-permeation of H_2_S which may subsequently act again as HS^−^ in the cytoplasm and be transferred in a post-translational modification to ion channels/transporters or related signaling molecules.

Aside from being a chemical environmental threat, H_2_S has recently been recognized as a gaseous signaling molecule which affects various physiological processes [26]. Due to its anti-inflammatory, anti-oxidant and vasoactive properties, H_2_S is currently evaluated as a potential therapeutic strategy in various models of lung diseases [27], [28], [29] including ALI/ARDS [4], [30], [31], [32], [33]. ALI/ARDS patients develop pulmonary edema and this correlates with the capacity of functional transepithelial Na^+^ absorption [11]. Both, beneficial and detrimental effects of H_2_S on edema formation have been reported in ALI models – depending on the employed concentration as well as whether H_2_S was inhaled or applied systemically [4]. The herein reported inhibition of Na^+^ absorption by H_2_S may account for the detrimental effects (including pulmonary edema) which have been observed upon exposure to higher concentrations of H_2_S (>50 ppm) in mice [4].

Taken together, inhibition of transepithelial Na^+^ transport provides a mechanism which may enhance edema formation in H_2_S exposed lungs. This finding expands the basic understanding of the pulmonary toxicology of H_2_S. Furthermore, the interference of H_2_S with ion transport processes in pulmonary epithelia needs to be carefully taken into consideration when exploring the therapeutic potential of H_2_S in the diseased lung.
